# The Gut Microbiota Regulates Endocrine Vitamin D Metabolism through Fibroblast Growth Factor 23

**DOI:** 10.3389/fimmu.2018.00408

**Published:** 2018-03-02

**Authors:** Stephanie A. Bora, Mary J. Kennett, Philip B. Smith, Andrew D. Patterson, Margherita T. Cantorna

**Affiliations:** ^1^Department of Veterinary and Biomedical Sciences, The Pennsylvania State University, University Park, PA, United States; ^2^The Huck Institutes of Life Sciences, The Pennsylvania State University, University Park, PA, United States; ^3^Eberly College of Science, The Pennsylvania State University, University Park, PA, United States

**Keywords:** microbiota, fibroblast growth factor 23, vitamin D, inflammation, tumor necrosis factor-α

## Abstract

To determine the effect of the microbiota on vitamin D metabolism, serum 25-hydroxyvitamin D(25D), 24,25-dihydroxyvitamin D (24,25D), and 1,25-dihydroxyvitamin D (1,25D) were measured in germ-free (GF) mice before and after conventionalization (CN). GF mice had low levels of 25D, 24,25D, and 1,25D and were hypocalcemic. CN of the GF mice with microbiota, for 2 weeks recovered 25D, 24,25D, and 1,25D levels. Females had more 25D and 24,25D than males both as GF mice and after CN. Introducing a limited number of commensals (eight commensals) increased 25D and 24,25D to the same extent as CN. Monocolonization with the enteric pathogen *Citrobacter rodentium* increased 25D and 24,25D, but the values only increased after 4 weeks of *C. rodentium* colonization when inflammation resolved. Fibroblast growth factor (FGF) 23 was extremely high in GF mice. CN resulted in an increase in TNF-α expression in the colon 2 days after CN that coincided with a reduction in FGF23 by 3 days that eventually normalized 25D, 24,25D, 1,25D at 1-week post-CN and reinstated calcium homeostasis. Neutralization of FGF23 in GF mice raised 1,25D, without CN, demonstrating that the high FGF23 levels were responsible for the low calcium and 1,25D in GF mice. The microbiota induce inflammation in the GF mice that inhibits FGF23 to eventually reinstate homeostasis that includes increased 25D, 24,25D, and 1,25D levels. The microbiota through FGF23 regulates vitamin D metabolism.

## Introduction

The gastrointestinal tract is colonized with complex communities of microorganisms that regulate host metabolism and immune function (1, 2). Germ-free (GF) mice are useful for determining the effects of microbes on host physiology. Experimental animal models of inflammatory bowel disease (IBD) fail to develop in GF mice (2–4). GF mice have underdeveloped immune systems and elevated Th2 responsiveness and reduced Th1/Th17 and Treg responses (2, 5, 6). Colonization of GF mice with microbes decreased the Th2 response, increased the Th1 and Th17 responses and induced regulatory T cells (7–9). The microbiome is an important regulator of immunity.

Vitamin D is either produced in the skin following UVB exposure, or consumed as part of the diet. Vitamin D is inactive and is hydroxylated twice to form the high affinity ligand for the vitamin D receptor (VDR), 1,25-dihydroxyvitamin D (1,25D). The vitamin D metabolizing enzymes are CypP450 genes. Vitamin D 25hydroxylation occurs in the liver, where Cyp2R1 and possibly Cyp27A1 catalyze the production of 25-hydroxyvitamin D (25D) (10). 25D is a relatively stable molecule, has a relatively long half-life, and reflects vitamin D intake (11). 25D is a low affinity ligand for the VDR, and under normal physiological conditions does not bind to the VDR (12). The vitamin D 1 α-hydroxylase (Cyp27B1) is expressed primarily in kidney and produces 1,25D (13). 1,25D binds to the VDR in nanomolar concentrations and acts as a transcription regulator (14). Cyp enzymes found in either the liver or kidney regulate endocrine vitamin D metabolism.

Vitamin D regulates calcium homeostasis and bone formation. The level of circulating 1,25D is tightly controlled by multiple interconnected feedback loops to maintain serum calcium levels in a small range (15, 16). When serum calcium is low, the parathyroid hormone (PTH) goes up and stimulates Cyp27B1 in the kidney to produce 1,25D (17). The increase in 1,25D inhibits PTH expression and induces the expression of the vitamin D 24hydroxylase (Cyp24A1). Cyp24A1 hydroxylates both 25D and 1,25D to form 24,25-dihydroxyvitamin D (24,25D) and 1,24,25-trihydroxyvitamin D, which leads to the clearance of 1,25D and a decreased pool of available 25D (18–21). Fibroblast growth factor (FGF) 23 inhibits PTH and induces Cyp24A1, which results in decreased 1,25D production (22). FGF23, PTH, and 1,25D form a series of feedback loops that together regulate 1,25D production to maintain mineral homeostasis.

Vitamin D is an important regulator of experimental IBD. Cyp27B1 knockout (KO) and VDR KO mice were extremely susceptible to colitis (23). The increased susceptibilities of Cyp27B1 KO and VDR KO mice to colitis were shown to be caused in part by shifts in the composition of the gut microbiota in mice (24). Sequencing of fecal DNA from Cyp27B1 KO and VDR KO mice showed increased frequencies of *Proteobacteria* and colitis causing *Helicobacteraceae* family members (24). Vitamin D supplementation in humans and 1,25D treatment of Cyp27B1 KO mice decreased *Proteobacteria* and increased beneficial organisms including members of the *Firmicutes* phyla (24, 25). Vitamin D regulates host immunity, experimental colitis, and the composition of the microbiota.

To determine whether the microbiota regulated vitamin D metabolism; serum levels of vitamin D, 25D, 24,25D, and 1,25D were measured in GF mice before and after conventionalization (CN). GF mice had high FGF23 with low 1,25D and 24,25D, and hypocalcemia. CN for 2 weeks increased 25D, 24,25D, and 1,25D levels significantly. Colonization with only commensals [altered Schaedler’s flora (ASF)], also raised 25D, and 24,25D levels in the previously GF mice. Monocolonization with a murine enteric pathogen raised 25D, and 24,25D levels, after 4 weeks, but not 2 weeks, of colonization. Neutralizing FGF23 in GF mice was effective for increasing 1,25D and 24,25D levels. Females had higher levels of 25D and 24,25D than males both when they were GF or CN. The earliest effects of CN were on inflammation and FGF23; that was followed by changes in calcium, 1,25D, and 24,25D. GF mice have defects in vitamin D metabolism that are corrected slowly following reduction in FGF23 and reinstatement of homeostasis which includes increased 1,25D.

## Materials and Methods

### Mice

Germ-free C57BL/6 wild-type (WT) mice were bred and maintained at The Pennsylvania State University (University Park, PA, USA) gnotobiotic animal research facility. The GF isolators and mice are routinely monitored to ensure the GF status of the mice. All mice were fed the autoclaved sterile chow routinely fed to maintain the GF colony. In addition, GF mice have enlarged cecums, which is additional assurance of their GF status on necropsy. At the start of the experiments, mice were 5–8 weeks of age. Male and female mice were used as indicated in the figure legends. Mice were orally supplemented with 5 µg vitamin D_3_ in corn oil, or vehicle treated with corn oil, three times weekly. Microbial transplantation was done using the cecal contents of WT mice. For some experiments stool from ASF (Taconic Biosciences, Hudson, NY, USA) colonized mice was used as the source of microbes for transplantation. Infection with pure strains of *Citrobacter rodentium* used strain ICC169 (gift of Gad Frankel, London School of Medicine and Dentistry, London, United Kingdom, nalidixic acid resistant) cultured in Luria-Bertani (EMD Chemicals, Inc., Gibbstown, NJ, USA) exactly as described (26). The monoclonization with *C. rodentium* was confirmed by doing gram stains of the cecal contents when the mice were sacrificed. All experimental procedures were approved by the Office of Research Protection’s Institutional Animal Care and Use Committee (Pennsylvania State University).

### LPS and FGF23 Neutralization

Mice were injected ip with 6 mg/kg LPS (Sigma-Aldrich, St. Louis, MO, USA) in sterile PBS and blood was collected 6 and 12 h later. For FGF23 neutralization, mice were injected ip with 5 mg/kg rat monocolonal anti-FGF23 or rat isotype control (kindly provided by Amgen, Thousand Oaks, CA, USA) three times weekly for 2 weeks, and sacrificed 24 h after the last injection.

### Vitamin D Metabolite Measurements

Sample preparation was done as described by Kaufmann et al. (27). 100 µL of pooled 13C_3_-vitamin D_3_ (100 ng/mL), d_3_-25D_3_ (100 ng/mL), and d_6_-24,25D_3_ (50 ng/mL) (Isosciences, King of Prussia, PA, USA) internal standard was added to 50 µL of sample. 50 µL of 0.1 M hydrochloric acid, 50 µL 0.2 M zinc sulfate, and 225 µL of 100% methanol were added to precipitate protein as described (27). Organic extraction was done by adding 350 µL *n*-hexanes, 350 µL MTBE (methyl tertiary butyl ether, Acros Organics, Geel, Belgium) and collecting the upper organic phase. Derivatization was done by redissolving the dried residue in 30 µL 4-[2-(6,7-dimethoxy-4-methyl-3,4-dihydroquinoxalinyl)ethyl]-1,2,4-triazoline-3,5-dione (DMEQ-TAD, 0.1 mg/mL in ethyl acetate Santa Cruz Biotechnology, Santa Cruz, CA, USA), drying, and the residue was dissolved in 30 µL 50/50 acetonitrile/water. All other LC–MS/MS solvents and reagents were Optima LC–MS grade (Fisher Scientific, Pittsburgh, PA, USA). The limit of detection was 1 ng/mL each of vitamin D, 25D, and 24,25D. Concentrations of each vitamin D metabolite were determined by calculating the ratio of the integrated peak areas of the metabolite and the relevant internal standard, compared with the values obtained from the standard curve.

Samples (5 µL) were separated by reverse phase HPLC using a Prominence 20 UFLCXR system (Shimadzu, Columbia, MD, USA) with a Waters (Milford, MA, USA) BEH Phenyl column (100 mm × 2.1 mm 1.7 µm particle size) and a flow rate of 250 µL/min. Solvents used were HPLC grade water with 0.1% formic acid and HPLC grade acetonitrile with 0.1% formic acid. The initial conditions were 70% water and 30% acetonitrile, increasing to 50% acetonitrile at 10 min, 90% acetonitrile at 12 min where it was held at 90% acetonitrile until 13 min before returning to the initial conditions. The eluate was delivered into a 5600 (QTOF) TripleTOF using a Duospray™ ion source (AB Sciex, Framingham, MA, USA). The capillary voltage was set at 5.5 kV in positive ion mode with a declustering potential of 80 V. The mass spectrometer was operated with a 250 ms TOF scan from 50 to 950 *m*/*z*, and 7 100 ms MS/MS product ion scans (*m*/*z* 730.5, 733.5, 746.5, 749.5, 762.5, and 768.5) from 50 to 950 per duty cycle using a collision energy of 45 V with a 30 V spread.

### 1 ,25D Measurements

1,25D was measured using a chemiluminescent assay or LC–MS. LIASON XL chemiluminescent assay was performed by Dr. Claudia Zierold (detection limit 5 pg/mL, DiaSorin, Stillwater, MN, USA) (28). For measurement by LC–MS, 1,25D was first concentrated according to kit instructions (Immundiagnostick, Bensheim, Germany) and measured as described earlier using authentic standards (d_3_-1,25D_3_ and 1,25D_3_, Isosciences) 1,25D samples (10 µL) were separated as described earlier with a Waters (Milford, MA, USA) BEH Phenyl column (150 mm × 1.0 mm 1.7 μm particle size) and a flow rate of 90 µL/min. The eluate was delivered either into a 5600 QTOF or a 6500 QTRAP as described earlier, with two 100 ms MS/MS product ion scans (*m*/*z* 762.5 and 765.5) from 50 to 950 per duty cycle using a collision energy of 37 V. For the QTRAP the capillary voltage was 5.5 kV in positive ion mode with a declustering potential of 80 V and the collision energy was 38 V. LC–MS/MS using the QTRAP was performed by Dr. Rahul Baghla (detection limit 4 pg/mL, Sciex, Redwood City, CA, USA). Chromatograms, ion spectra, structure, and fragmentation of DMEQ-TAD 4-[2-(6,7-dimethoxy-4-methyl-3,4-dihydroquinoxalinyl)ethyl]-1,2,4-triazoline-3,5-dione (DMEQ-TAD) adducts of vitamin D, 25D, 24,25D, and 1,25D examples are shown in Figure S1 in Supplementary Material (27).

### PTH, FGF23, and Calcium Measurements

Parathyroid hormone (1–84) and FGF23 were measured by ELISA, according to manufacturer’s instructions (Immutopics, San Clemente, CA, USA). Limits of detection were 32 pg/mL PTH and 25 pg/mL FGF23. Serum calcium levels were measured by colorimetric assay using QuantiChrom Calcium Assay Kit (BioAssay Systems, Hayward, CA, USA), according to manufacturer’s instructions.

### RNA Isolation and RT-PCR

RNA from kidney, liver, and colon were extracted using TRIzol reagent using manufacturer’s instructions. 4 μg/sample RNA was reverse transcribed into cDNA using AMV reverse transcriptase (Promega, Madison, WI, USA). Mouse HPRT, Cyp2R1, *cyp27a1, cyp24a1, cyp27b1, vdr, tnf*-α, *il-1*β, *il-6*, and *ifn-*γ mRNA were quantified by real-time PCR using the StepOnePlus real-time PCR system (Thermo Fisher Scientific, Rockford, IL, USA) with StepOnePlus software and BioRad SYBR Green Master Mix (Hercules, CA, USA). Gene expression was determined as relative expression compared with a linear curve based on a gel-extracted standard. Values were normalized to *hprt* and expressed as a fold change over GF. Primer sequences can be found in Table S1 in Supplementary Material.

### Colon Histology

Distal colon was fixed in 10% formalin, sectioned, and stained with hematoxylin and eosin (Pennsylvania State University Animal Diagnostic Laboratory). Sections were scored blinded by a board-certified laboratory animal veterinarian with training in pathology (Dr. Mary Kennett) and scored on a scale from 0 to 3 for extent of inflammatory cell infiltrates, and from 0 to 4 for severity of inflammatory cell infiltrates. Values of 0 = normal, 1 = mucosal, 2 = mucosal and submucosal, and 3 = mucosal, submucosal, and transmural for extent of inflammatory cell infiltrates. Values of 0 = none, 1 = minimal, 2 = mild, 3 = moderate, and 4 = marked for severity of inflammatory cell infiltration. Scores were added, resulting in a total inflammation score of 7 for each sample.

### Statistical Analysis

Statistical analyses were performed using GraphPad Prism software (GraphPad, La Jolla, CA, USA). One-way ANOVA with Tukey’s *post hoc* and two-way ANOVA with Bonferroni’s *post hoc* test were used to compare levels of vitamin D metabolites, PTH, and FGF23 in mice. Two-tailed Student’s *t*-test was used for some vitamin D metabolite, FGF23, and calcium measurements. For all analyses, * indicates *P* < 0.05, ** indicates *P* < 0.01, and *** indicates *P* < 0.0001.

## Results

### Increased 25D and 24,25D Values following CN of GF Mice

**Figure 1:**
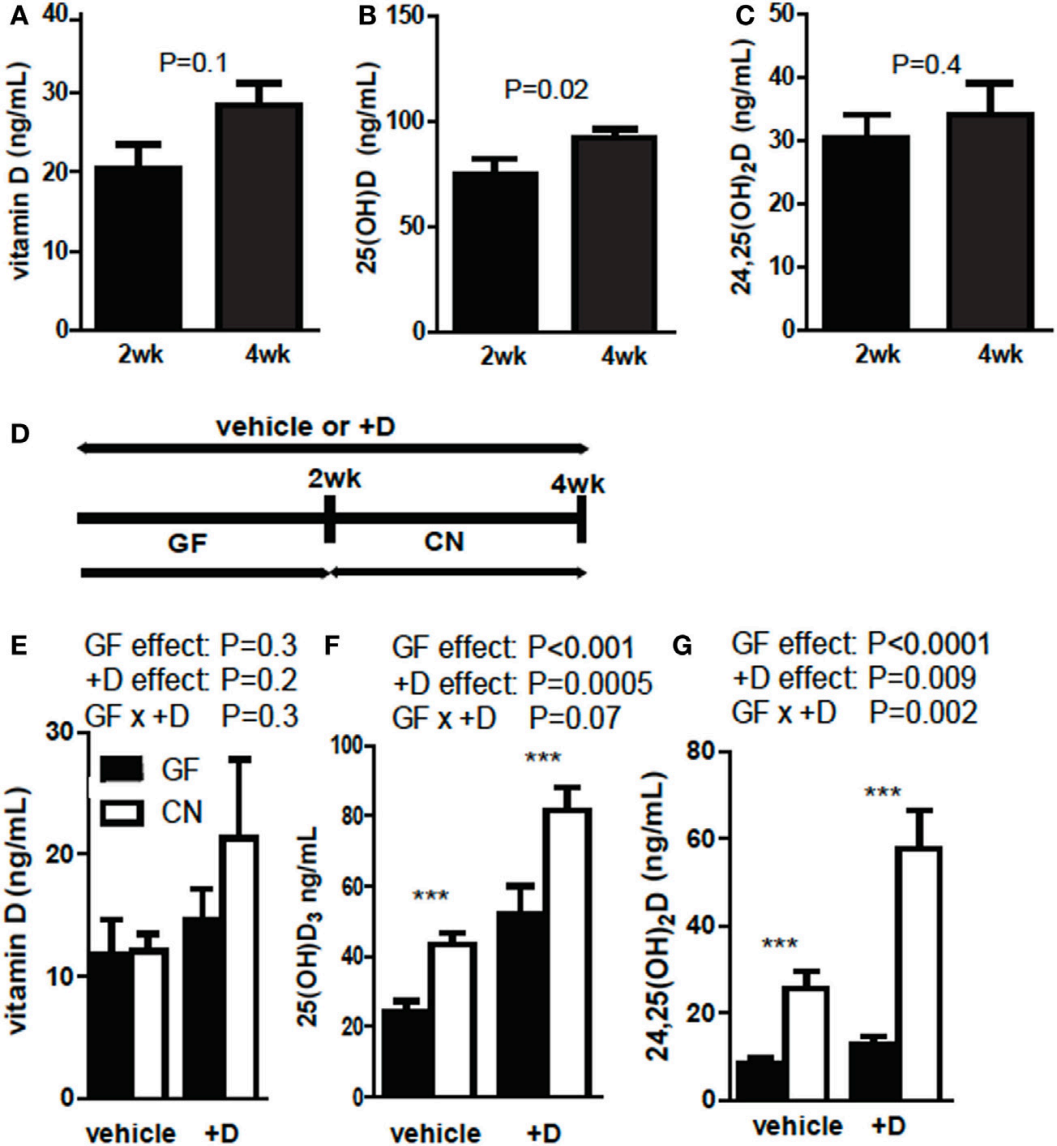
25D and 24,25D increases following CN of germ-free (GF) mice. **(A)** Vitamin D, **(B)** 25D, and **(C)** 24,25D at 2 and 4 weeks of +D supplementation. Values are ± SEM of *n* = 10 mice per group, five males and five females. Student’s *t*-test was used to test significance. **(D)** Experimental design of conventionalization (CN) and vehicle or +D supplementation. **(E)** Vitamin D, **(F)** 25D, and **(G)** 24,25D levels from plasma of GF before and after 2-week CN. Values are the mean ± SEM of *n* = 9 mice per group, four males and five females, and two independent experiments. Two-way ANOVA with Bonferroni *post hoc* tests was used to test significance (*P < 0.05, **P < 0.01, and ***P < 0.001).

Germ-free mice were supplemented with vitamin D for 2 and 4 weeks and vitamin D, 25D, and 24,25D levels were measured in the +D GF mice. Vitamin D levels (*P* = 0.1, Figure 1A) and 24,25D levels (*P* = 0.4, Figure 1C) were not different after 2 or 4 weeks of +D supplementation of GF mice. 25D levels were significantly lower at 2 weeks of +D supplementation than after 4 weeks of +D supplementation of GF mice (*P* = 0.02, Figure 1B). GF mice were vehicle treated or +D supplemented for 4 weeks (Figure 1D) as above. After 2 weeks of +D or vehicle treatment the GF mice were bled and then CN, while maintaining the vehicle and +D treatments (Figures 1E–G). The vitamin D metabolites were then measured in the mice before and after CN (Figures 1E–G). Vitamin D levels were not affected by either the vitamin D supplementation or CN (Figure 1E). The levels of 25D increased significantly in both vehicle and +D treated mice after CN (*P* < 0.0001, Figure 1F). The increase in 25D seen following CN of vehicle treated mice suggests that the microbiota have additional effects on 25D levels outside of any accumulation seen following the 4-week +D treatment (Figures 1B,F). +D mice had higher serum 25D compared with mice receiving vehicle (*P* = 0.0005), though the combination of +D and CN resulted in the greatest increase in 25D (Figure 1F). There was not a significant interaction between +D and CN on serum 25D levels (*P* = 0.07, Figure 1F). There was no change in 24,25D levels between vehicle (12 ng/mL) and +D (13 ng/mL) treated GF mice. CN caused an overall significant increase in 24,25D in vehicle and +D mice (*P* < 0.0001). There was an effect of +D supplementation on serum 24,25D levels (*P* = 0.009, Figure 1G). There was a significant interaction between +D and CN on 24,25D levels in the serum of mice (*P* = 0.002, Figure 1G). +D mice had the highest 25D and 24,25D levels following CN and therefore the rest of the experiments were done using +D treated mice.

**Figure 2:**
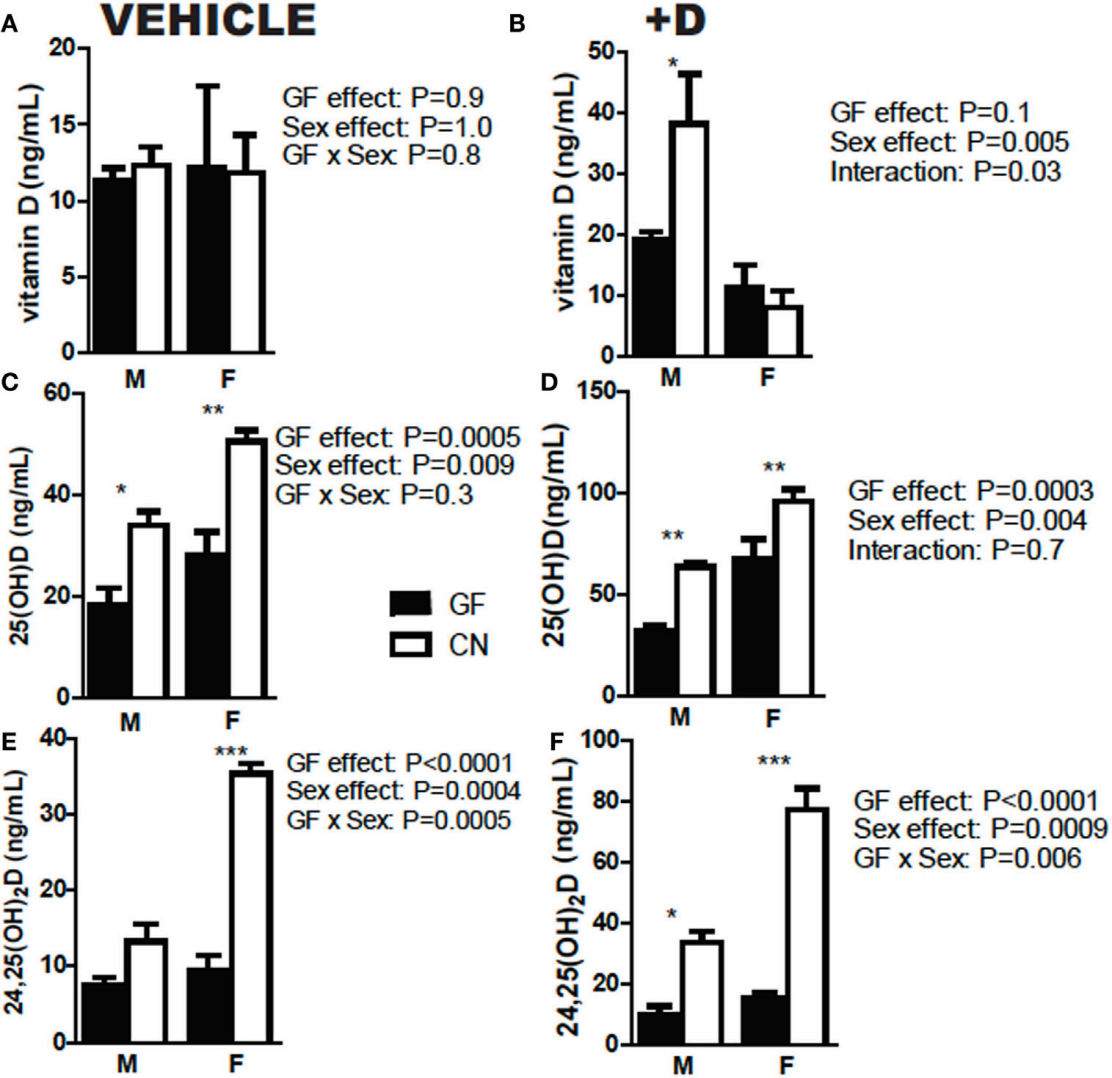
Females produce more 25D and 24,25D than males. **(A,B)** Vitamin D, **(C,D)** 25D, and **(E,F)** 24,25D in vehicle and +D, male and female mice, before and after 2 weeks conventionalization. Values are the mean ± SEM of *n* = 9 mice per group, four males and five females, and two independent experiments. Two-way ANOVA with Bonferroni *post hoc* tests was used to test significance (*P < 0.05, **P < 0.01, and ***P < 0.001).

The experiments shown in Figure 1 used both male and female mice. Sex was also evaluated separately for effects on vitamin D metabolism (Figure 2). +D males had higher vitamin D levels following CN than females (Figure 2B). Overall, female mice produced more 25D than male mice (Figures 2C,D). In addition, female mice had the biggest increase in both 25D and 24,25D following CN (Figures 2C,D). There were significant effects of CN in both +D male and +D female mice for 25D and 24,25D levels (Figures 2D,F). Females produced more 25D and 24,25D than males (Figures 2C–F). Since sex is an important variable affecting the measurements of vitamin D metabolites, experiments used equal numbers of males and females when possible, or only one sex as indicated in each figure.

### The Effects of CN on Expression of mRNA for Cyp Metabolizing Enzymes

mRNA for *cyp2R1, cyp27A1, cyp24A1, cyp27B1*, and the *vdr* were measured in GF mice and 2-week CN mice. mRNA for *cyp2r1* (*P* = 0.004) and *cyp27a1* (*P* = 0.04) increased in the liver following CN (Figure S2A in Supplementary Material). In the kidney *cyp24a1* was significantly inhibited (*P* = 0.009, Figure S2B in Supplementary Material) and *cyp27b1* was significantly increased following CN (*P* = 0.03, Figure S2B in Supplementary Material). There was no change in kidney expression of *vdr* mRNA following CN of GF mice (Figure S2B in Supplementary Material). In the colon, CN resulted in an insignificant induction of *cyp24a1* (*P* = 0.06), no effect on *cyp27b1*, and significant inhibition of *vdr* expression (Figure S2C in Supplementary Material). CN resulted in increased mRNA in the liver for Cyp2R1 and Cyp27A1 that corresponded with the increase in 25D following CN. In the kidney, expression of mRNA for *cyp24a1* went down at 2 weeks post-CN, even though 24,25D levels were higher in the serum of CN mice suggesting post-transcriptional regulation of *cyp24a1*. Expression of *cyp27b1* was higher in the kidney and VDR was higher in the colon with CN.

### Kinetics of Increased 24,25D following CN of GF Mice

**Figure 3:**
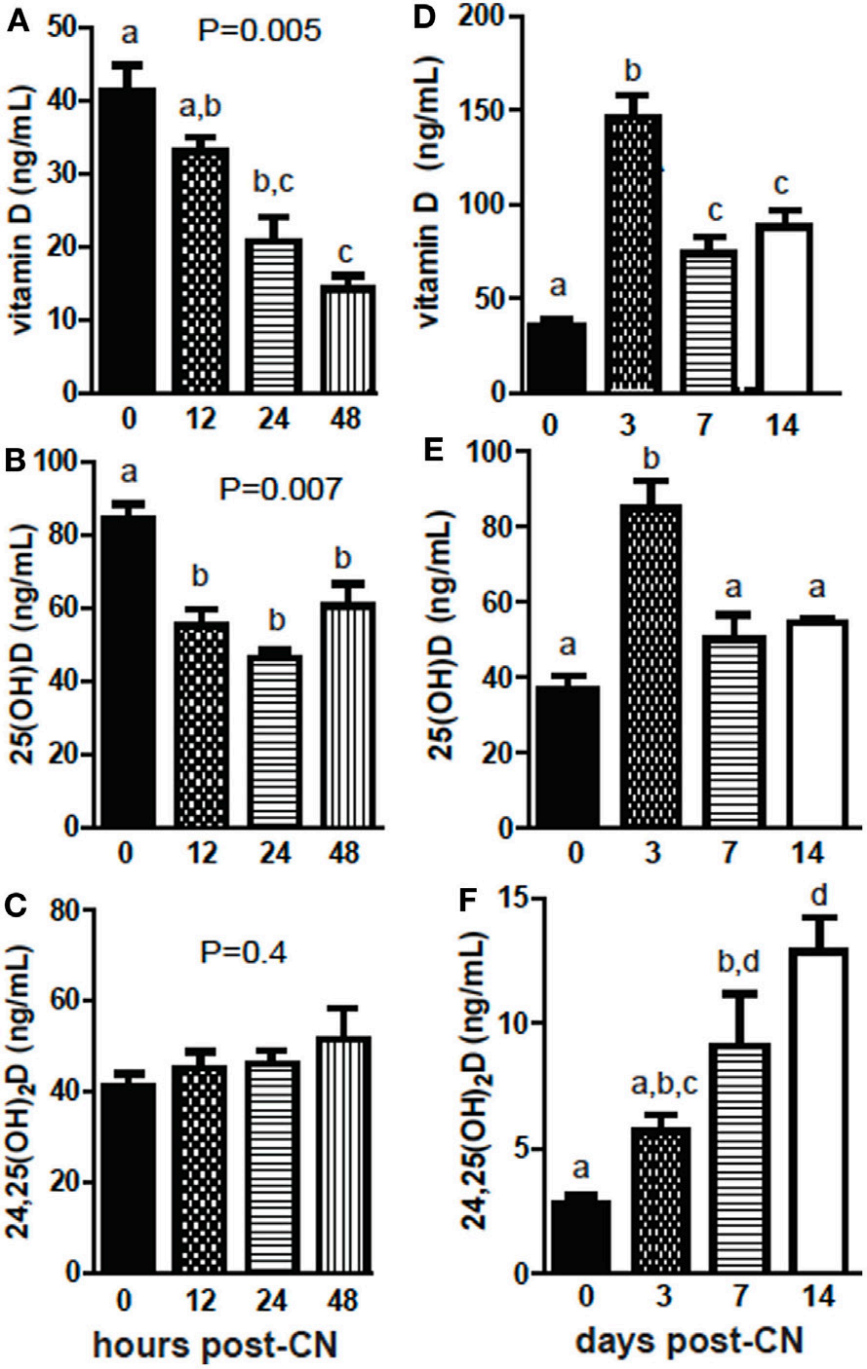
The kinetics of increased 24,25D following conventionalization (CN) of germ-free mice. **(A)** Vitamin D, **(B)** 25D, and **(C)** 24,25D from 0 to 48 h after CN. Values are the mean ± SEM of *n* = 3–4 males per group. **(D)** Vitamin D, **(E)** 25D, and **(F)** 24,25D from 0 to 2 weeks after CN. Values are the mean ± SEM of *n* = 5–9 female mice per group. One-way ANOVA with Tukey’s *post hoc* tests was used to test significance. Bars without a letter in common indicate a significant difference.

+D GF mice were dosed and sacrificed at several different time points following CN to determine the kinetics of the increases in 25D and 24,25D. Early following CN, vitamin D levels decreased and remained low 48 h post-CN (*P* = 0.005, Figure 3A). The reduced vitamin D was accompanied by reduced levels of 25D (*P* = 0.007, Figure 3B). 24,25D levels went up early post-CN compared with GF mice, but did not reach significance at 48 h post-CN (*P* = 0.4, Figure 3C). Vitamin D levels increased significantly at 3 days and were higher at 14-day post-CN than in GF female mice (*P* < 0.0001, Figure 3D). 25D levels in females spiked at 3 days and remained higher (insignificant) than GF mice at 14-day post-CN (*P* < 0.0001, Figure 3E). 24,25D levels increased in female (*P* < 0.0001, Figure 3B) and mixed sexes (*P* = 0.02) following CN (data not shown), and the increase in 24,25D was significantly higher by 7d of CN (Figure 3F). The more frequent collection of blood needed for the kinetic experiments shown in Figure 3 required sacrificing mice at different time points. The data shown in Figures 1 and 2 utilized the same mice before and after CN. This may account for the discrepancies between the effects of CN on vitamin D and 25D measurements between experiments. Nonetheless, the data are consistent for the increased 24,25D levels as a result of CN. The effects of the microbiota on 24,25D levels were evident by d7 of CN.

### Colonization with Only Commensals Increased 24,25D

**Figure 4:**
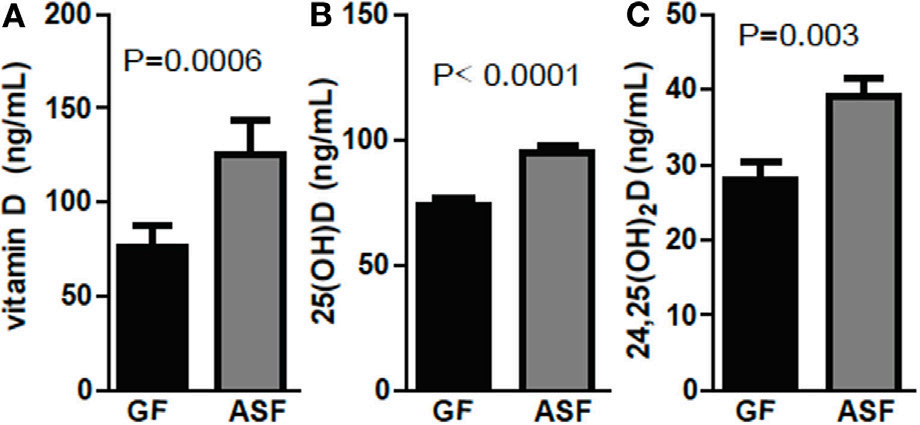
Colonization with commensals increased 24,25D after 2 weeks. **(A)** Vitamin D, **(B)** 25D, and **(C)** 24,25D levels following colonization of germ-free (GF) mice with altered Schaedler’s flora (ASF). Values are the mean ± SEM of *n* = 10–15 mice per group, 5 males and 10 females, and 2 independent experiments. Student’s *t*-test was used to test significance.

Conventionalization of GF mice increased 24,25D levels significantly across multiple experiments (Figures 1–3). ASF, which contains eight commensal organisms, was used to colonize GF mice (29, 30). Colonization of +D GF mice with ASF significantly increased vitamin D, 25D, and 24,25D levels in the serum (Figure 4). Vitamin D increased with ASF colonization at 2 weeks (*P* = 0.0006 Figure 4A). Increased vitamin D levels with CN have been seen in some experiments (Figure 3D) but not all (Figure 1). ASF colonization for 2 weeks increased 25D levels significantly (*P* < 0.0001, Figure 4B) and increased 24,25D levels significantly (*P* = 0.003, Figure 4C).

### Monocolonization with *C. rodentium* Increased 24,25D after 4 weeks

**Figure 5:**
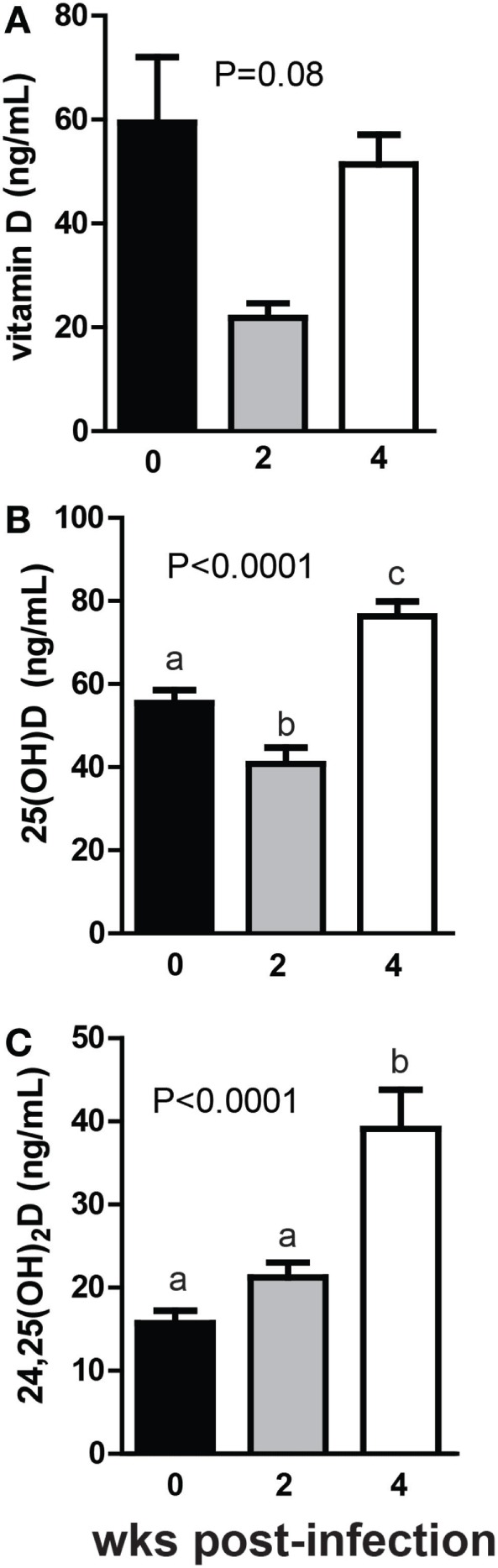
Monocolonization with *Citrobacter rodentium* increased 24,25D after 4 weeks. **(A)** Vitamin D, **(B)** 25D, and **(C)** 24,25D at 0, 2, and 4 weeks after colonization with 1 × 10^9^ CFU *C. rodentium*. Values are the mean ± SEM of *n* = 7–16 mice per group, 6 males and 10 females, and 2 independent experiments. One-way ANOVA with Tukey’s *post hoc* tests was used to test significance. Bars without a letter in common indicate a significant difference.

+D GF mice were infected with *C. rodentium*, a murine enteric pathogen. *C. rodentium* infection of WT mice is cleared within 4 weeks of colonization, while in GF mice the infection is not cleared although the mice no longer exhibit symptoms [inflammation or diarrhea (31)]. Vitamin D levels went down in GF mice monocolonized with *C. rodentium* for 2 weeks and then back up after 4 weeks of monocolonization (Figure 5A). The changes in vitamin D over time following monocolonization with *C. rodentium* were not significant (*P* = 0.08, Figure 5A). There was a decrease in 25D at 2 weeks post-colonization with *C. rodentium* that mirrored the decline in vitamin D at this time point (*P* < 0.0001, Figure 5B). 25D levels increased significantly at 4 weeks of monocolonization compared with the levels in the GF mice before *C. rodentium* infection (*P* < 0.0001, Figure 5B). There was an insignificant increase in 24,25D at 2 weeks and a significant increase at 4 weeks of monocolonization with *C. rodentium* (*P* < 0.001, Figure 5C). Monocolonization with *C. rodentium* for 2 weeks decreased 25D levels and had no effect on 24,25D levels. The effects of *C. rodentium* monocolonization for 4 weeks were to increase 25D and 24,25D levels significantly compared with GF mice before infection (Figure 5).

### Effects of CN on Calcium, FGF23, and 1,25D Levels

**Figure 6:**
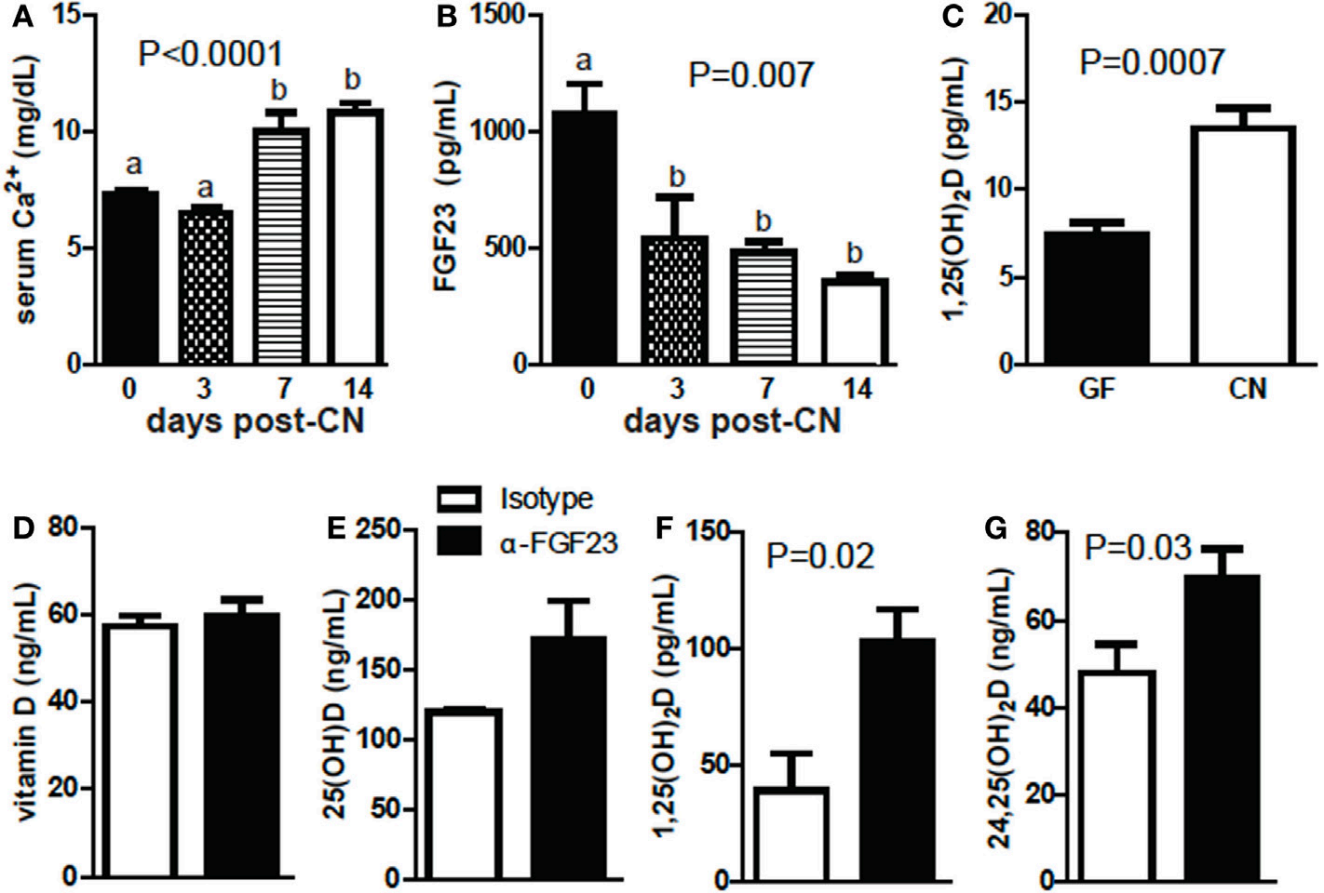
Conventionalization (CN) decreases fibroblast growth factor (FGF) 23 and increases calcium and 1,25D levels. **(A)** Serum Ca following CN of mice. Values are the mean ± SEM of *n* = 3–21 mice per group, and one to three independent experiments. **(B)** FGF23 levels following CN of mice. Values are the mean ± SEM of *n* = 3–6 female mice per group, and one to two experiments. One-way ANOVA with Tukey’s *post hoc* tests was used to test significance. Bars without a letter in common indicate a significant difference. **(C)** 1,25D levels following CN of previously germ-free (GF) mice. Values are the mean ± SEM of *n* = 7 female mice per group, from three independent experiments. **(D)** Vitamin D, **(E)** 25D, **(F)** 1,25D, and **(G)** 24,25D in GF mice receiving isotype or FGF23-neutralizing antibody. Values are the mean ± SEM of *n* = 6 mice per group, three males and three females. Student’s *t*-test was used to test significance.

Germ-free mice were hypocalcemic and had high levels of FGF23 and low levels of 1,25D (Figures 6A–C). PTH levels were not significantly different (data not shown). Calcium levels were low in the serum of mice 3-day post-CN and then were significantly higher at 1-week post-CN (*P* < 0.0001, Figure 6A). FGF23 levels went down 3-day post-CN and remained lower than GF values at 1- and 2-week post-CN (*P* = 0.007, Figure 6B). 1,25D levels were extremely low in GF and 3-day post-CN mice and in some experiments remained below or at detection levels (<5 pg/mL, Figure 6C; Figure S3 in Supplementary Material). 1,25D levels increased and were detectable in all mice by 1 week after CN (Figure S3 in Supplementary Material). At 2-week post-CN mice had significantly higher 1,25D levels than GF mice (*P* = 0.007, Figure 6C). FGF23 blocking antibodies were administered to +D GF mice. Blocking FGF23 in +D GF mice had no effect on vitamin D or 25D levels (Figures 6D,E) but significantly increased 1,25D (*P* = 0.02, Figure 6F) and 24,25D levels (*P* = 0.03, Figure 6G). Either CN or blocking FGF23 in GF mice induced 1,25D and 24,25D levels.

### The Effect of CN on Colonic Inflammation

**Figure 7:**
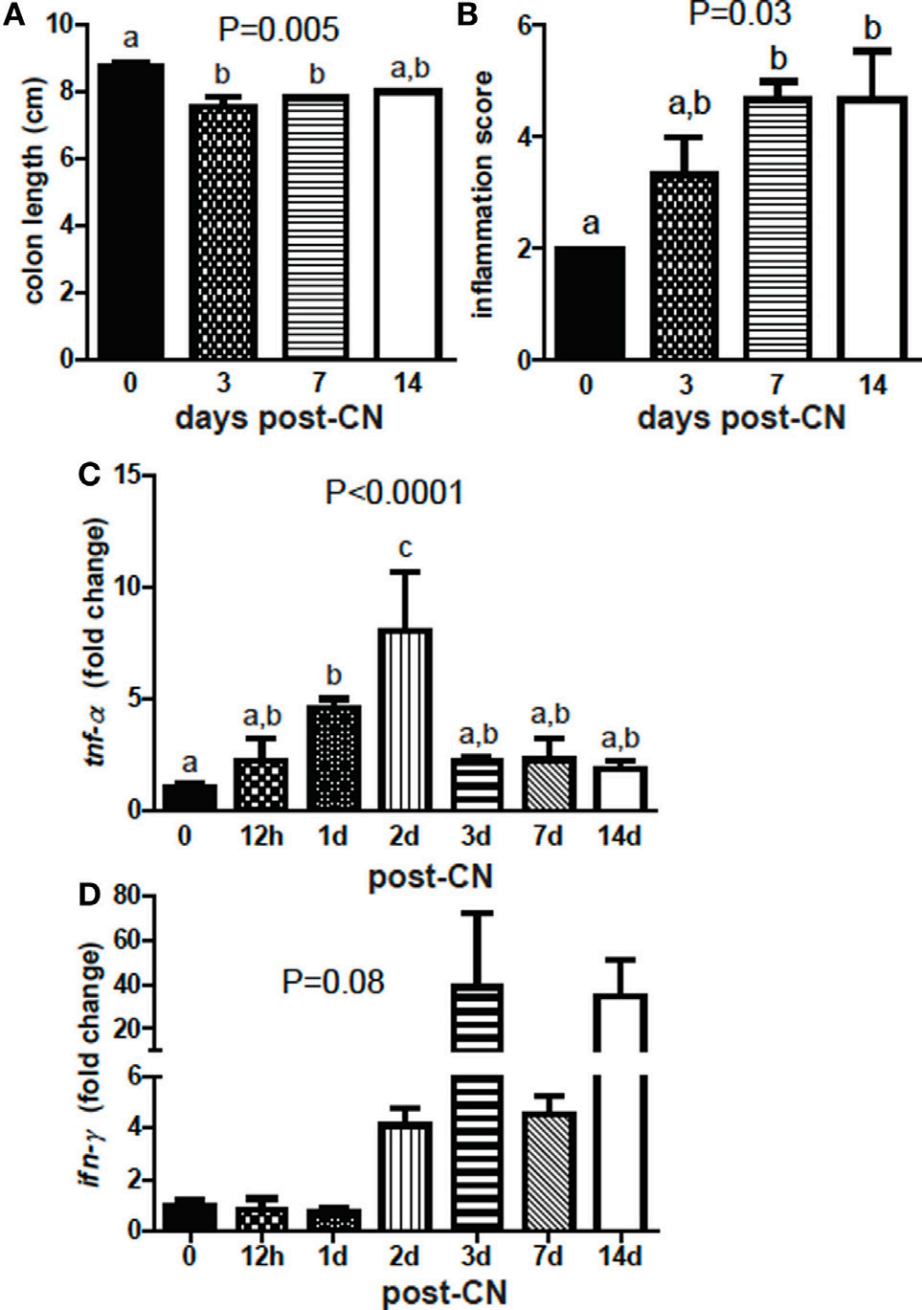
Conventionalization (CN) induces colonic shortening and TNF-α. **(A)** Colon length and **(B)** histological score of inflammation in distal colons from mice following CN. Values are the mean ± SEM of *n* = 3 females per group. **(C)** Expression of **(C)** *tnf-*α and **(D)** *ifn-*γ in the colon. Values are the mean ± SEM of *n* = 3–6 mice per group, 15 males and 6 females, and 1–2 independent experiments. One-way ANOVA with Tukey’s *post hoc* tests was used to test significance. Bars without a letter in common indicate a significant difference.

The colon length of GF mice decreased at day 3 of CN and did not change significantly thereafter (*P* = 0.005, Figure 7A). Histopathology sections showed an increase in inflammation in the colon (*P* = 0.03, Figure 7B). Histology scores were higher at 1 week of CN and remained unchanged at 2 weeks compared with GF histology scores (Figure 7B). mRNA for *tnf-*α increased significantly after day 1 and was highest at day 2 of CN (*P* < 0.0001, Figure 7C). By day 3 of CN *tnf-*α mRNA went down to the same level present in GF mice (Figure 7C). mRNA in the colon for *il-1*β, and *il-6* did not change significantly with CN (data not shown). *ifn-*γ mRNA was higher but not significantly higher with time post-CN (*P* = 0.08, Figure 7D). CN resulted in early (day 1–day 2 post-CN) expression of *tnf-*α mRNA in the colon and shortening of the colon compared with GF mice.

### Endotoxemia Reduced 24,25D

**Figure 8:**
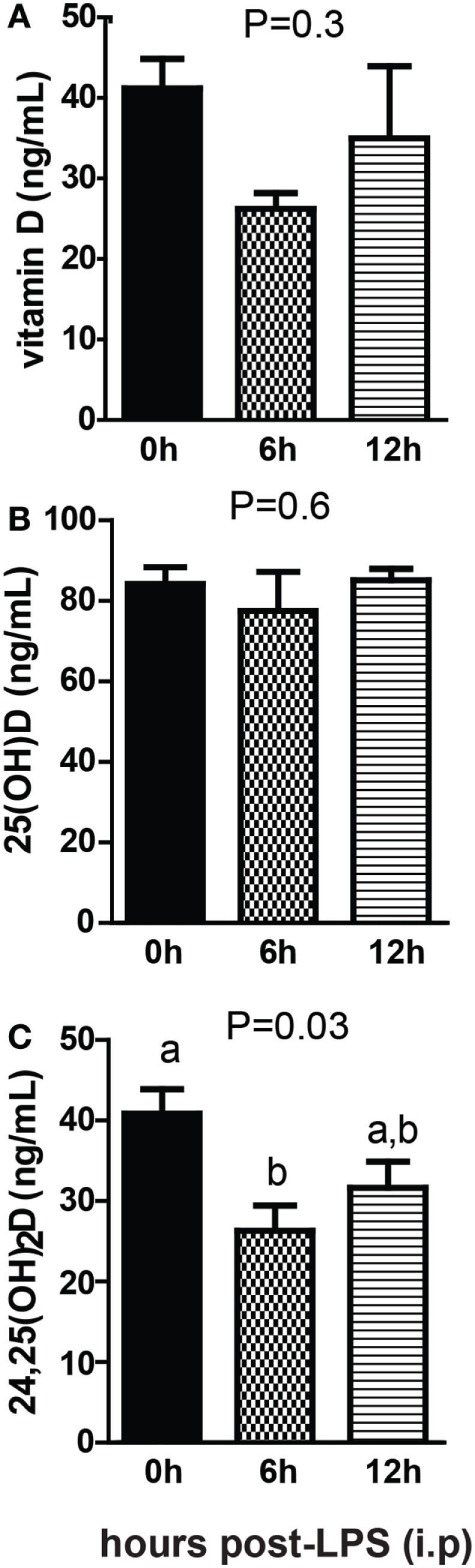
Sterile inflammation reduces 24,25D levels. **(A)** Vitamin D, **(B)** 25D, and **(C)** 24,25D from +D germ-free, 6 and 12 h after LPS injection. Values are the mean ± SEM of *n* = 4–5 males per group. One-way ANOVA with Tukey’s *post hoc* tests was used to test significance. Bars without a letter in common indicate a significant difference.

Sterile inflammation was induced in +D GF mice following injection with LPS. There was no significant change in vitamin D after LPS injection (Figure 8A) There was no effect of LPS injection on 25D levels (Figure 8B). 24,25D decreased significantly after LPS injection, with the largest decrease 6 h after injection (*P* = 0.03, Figure 8C). Sterile inflammation reduced 24,25D levels in GF mice.

## Discussion

Germ-free mice have elevated FGF23, hypocalcemia, and low 1,25D and 24,25D levels. Neutralizing FGF23 increased 1,25D and 24,25D in GF mice, providing evidence that elevated FGF23 is the cause of the low levels of 1,25D and 24,25D in the GF host. FGF23 is produced primarily by bone-mineralizing osteoblasts and osteocytes, although expression of *fgf23* has been found in activated monocyte and dendritic cells (32, 33). GF mice have dense bones and fewer bone-resorbing osteoclasts than CN mice (34). CN of the GF mice normalized bone density and induced more T cells and osteoclasts in the bone marrow (34). LPS induced osteoclast development *via* TNF-α produced by bone marrow macrophages (35). Therefore, the dense bones found in GF mice are likely the cause of the extremely high FGF23. CN resulted in an immune mediated response to the microbiota as shown by d3 colonic shortening, histology, and early TNF-α response in the colon. The inflammatory perturbation that occurs following CN of GF mice resulted in the induction of osteoclasts that coincided with decreased FGF23. The late effects of CN on 1,25D, 24,25D and serum calcium suggest that the increase in 1,25D and 24,25D levels is a result of the reinstatement of homeostasis following normalization of FGF23.

Colonization of GF mice with only eight commensals (ASF) was the same as CN with complex microbial communities and resulted in increased 25D and 24,25D levels. The lack of differentiation between the commensals and complex microbial signals suggest that the host was responding to general bacterial signals, and not that a specific microbe is directing host vitamin D metabolism. The data might also indicate that the commensals are present in the complex microbial communities and provide the signals needed to suppress FGF23 and reinstate vitamin D homeostasis. Infection of GF mice with the murine pathogen *C. rodentium* decreased vitamin D and 25D levels at 2 weeks post-infection, which was likely due to decreased absorption of vitamin D while the infected mice had diarrhea. The peak of infection occurs after 1–2 weeks of *C. rodentium* infection and at that time there is significant inflammation and cytokine production, which resolves in both GF and CN mice by 4 weeks of infection (31). The *C. rodentium* pathogen induces significantly more damage to the GI tract than CN of GF mice. Once the inflammation in the GI tract resolves homeostasis is reinstated and 25D and 24,25D levels increased at 4 weeks following *C. rodentium* infection. Sterile inflammation following injection of GF mice with LPS significantly decreased 24,25D after 6 h. The effect of systemic inflammation is different than mucosal inflammation on the production of 24,25D. The very fast decrease in 24,25D levels following LPS injection suggests that Cyp24A1 may be directly suppressed *via* the LPS receptor (toll like receptor-4) or cytokine (TNF-α) signals, but this needs to be investigated further. Microbial regulation of vitamin D metabolism occurs at different rates that depend on the degree of inflammation.

Conventionalization increased 24,25D in both females and males, but females had a 2- to 2.5-fold higher increase following CN compared with males in both +D and vehicle groups. It is well known that sex hormones have effects on the microbiome (36–38). However, female/male microbial differences are probably not the cause of higher 25D and 24,25D levels in females, since ASF and CN had the same effect on 25D and 24,25D levels in both males and females. Instead the higher 25D and 24,25D might reflect more robust immune responses in females versus males, and the well described effects of estrogen on adaptive immunity (39). Estrogen and the sex effect on the immune response affect the composition of the microbiota in females (40, 41). There are also interactions between 1,25D/VDR and estradiol in females (42). Estradiol increased expression of the VDR and decreased expression of Cyp24A1 (42) suggesting direct regulation of vitamin D metabolism by estrogen. Increased 25D and 24,25D levels in female versus male mice may be due to the increased adaptive immune response in females and direct regulation of 25D and 24,25D levels by estrogen.

In the absence of the microbiota GF mice have high FGF23, and low levels of 25D, 24,25D, 1,25D, and calcium. Perturbations of homeostasis in the GF mouse resulted in the induction of an immune response that over time resulted in the reinstatement of homeostasis. In the GF mouse, the earliest changes are to the immune system and reductions in FGF23. Reduced FGF23 eventually reinstated vitamin D homeostasis, which included higher basal levels of calcium, 25D, 24,25D, and 1,25D. Disruptions in the microbiota and homeostasis of the gastrointestinal tract are associated with IBD (23). With more severe inflammation that occurs following *C. rodentium* infection at 2 weeks, vitamin D and 25D levels were lower. Patients with active Crohn’s disease have lower 25D levels, which is consistent with our data (43). As the inflammation in the colon resolved the 24,25D levels went up in the *C. rodentium* infected GF mice. Disruption of the microbiota and inflammation had important and previously unappreciated effects on the availability of 25D and 1,25D to the host.

## Ethics Statement

All experimental procedures were approved by the Office of Research Protection’s Institutional Animal Care and Use Committee (Pennsylvania State University).

## Author Contributions

SB designed and did the experiments, wrote the manuscript. MC designed experiments, wrote the manuscript, interpreted the results, and funded the work. MK evaluated histopathology and interpreted results. PS aided in experiment design and data interpretation. AP designed experiments and interpreted results.

## Conflict of Interest Statement

The authors declare that the research was conducted in the absence of any commercial or financial relationships that could be construed as a potential conflict of interest. The reviewer NT-J and handling editor declared their shared affiliation.
