# Supplementary material for: The Gut Microbiota Regulates Endocrine Vitamin D Metabolism through Fibroblast Growth Factor 23
Source: Front Immunol. 2018 Mar 2;9:408. doi: 10.3389/fimmu.2018.00408 (PMC5863497; doi:10.3389/fimmu.2018.00408)
Supplement: Supplementary file 1 [file data_sheet_1.docx]

**Supplementary Table 1. RT-PCR primer sequences.**

| HPRT | F: 5’-CAG ACT GAA GAG CTA TGG TAA TG-3’  R: 5’ CCA GTG TCA ATT ATA TCT TCC AC-3’ |
| --- | --- |
| Cyp2R1 | F: 5’-TTT GTC AAC ATC TGC T-3’  R: 5’-TGC CTC CAA GAT CTA AAC TGA AA-3’ |
| Cyp27A1 | F: 5’-CCT CAC CTA TGG GAT CTT CAT C-3’  R: 5’-TTT AAC GCA TCC GTG TAG AGC-3’ |
| Cyp24A1 | F: 5’-ACC CCC AAG GTC CGT GAC ATC-3’  R: 5’-CCA GTT GGG TCC AGG TAA GG-3’ |
| Cyp27B1 | F: 5’-GAC CAT ATT GGC CCG TAC C-3’  R: 5’-CAG CTT GGC TGA ACT CT-3’ |
| VDR | F: 5’-CTG CAC CTC CTC ATC TGT GA-3’  R: 5’-CCC CTT CAA TGG AGA TTG C-3’ |
| TNF-α | F: 5’-TGG GAG TAG ACA AGG TAC AAC CC-3’  R: 5’-CAT CTT CTC AAA ATT CGA GTG ACA A-3’ |
| IL-1β | F: 5’-AGT TGA CGG ACC CCA AAA G-3’  R: 5’-AGC TGG ATC CTC TCA TCA GG-3’ |
| IL-6 | F: 5’-GCT ACC AAA CTG GAT ATA ATC AGG A-3’  R: 5’-CCA GGT AGC TAT GGT ACT CCA GAA-3’ |
| IFN-γ | F: 5’-TGC ATC TTG GCT TGG CAG CTC TTC-3’  R: 5’-GGG TTG ACC TCA AAC TTG GCA-3’ |

**Supplementary Figure 1**

**
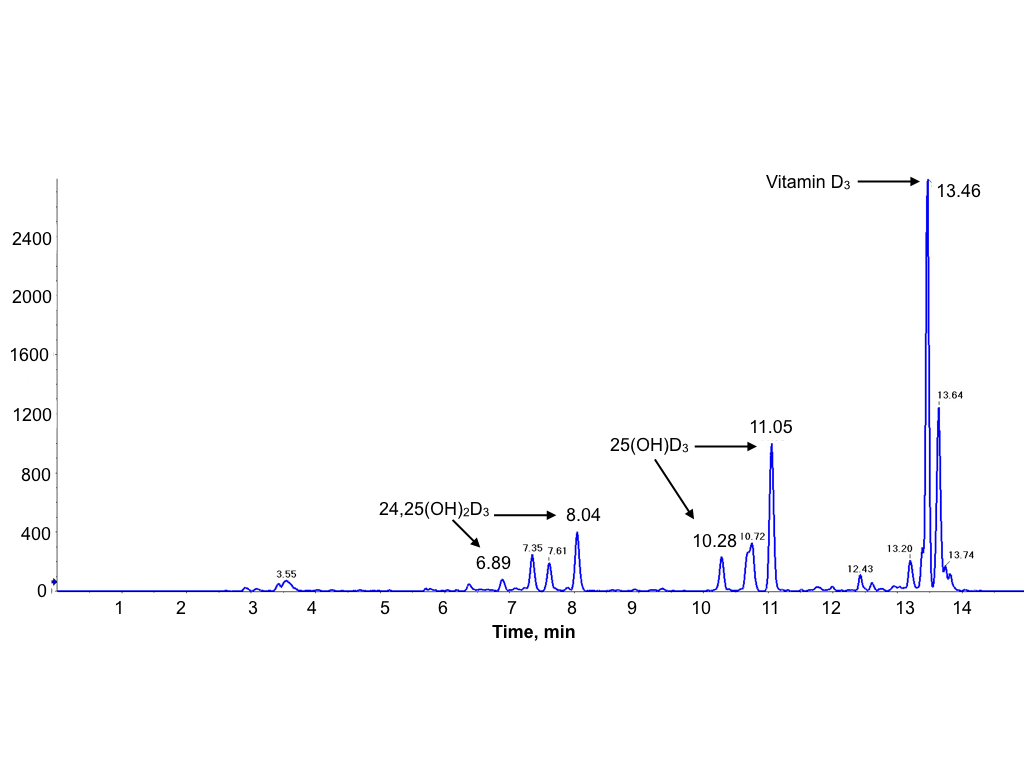
A**

**B**

**
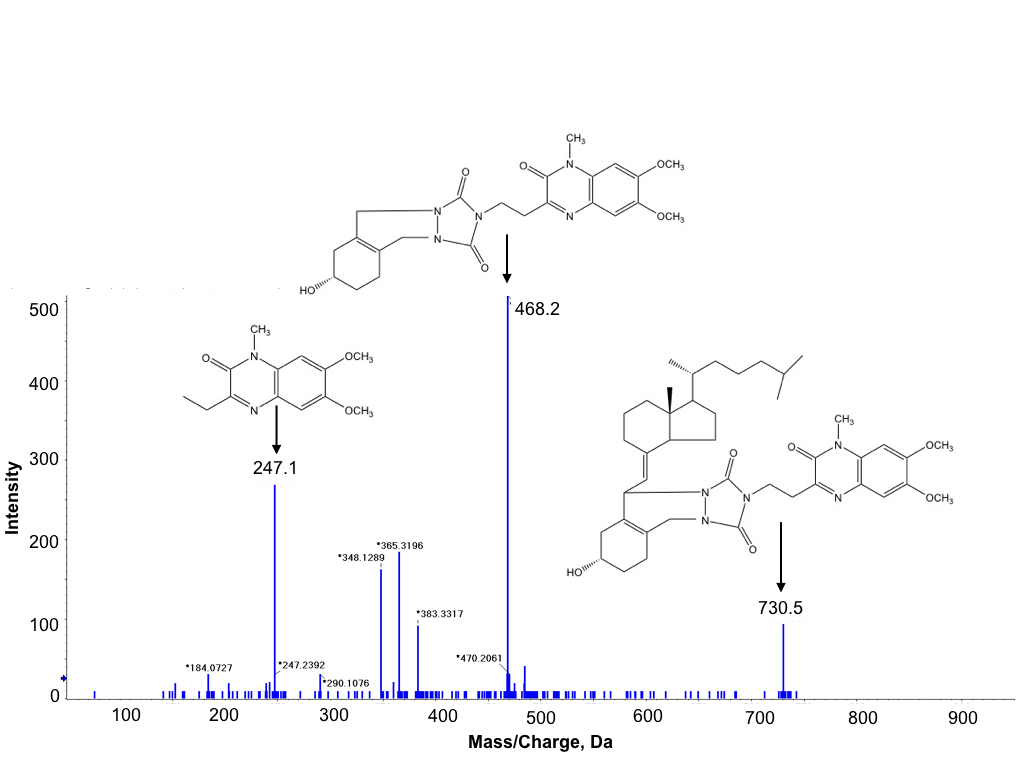
**

**Supplementary Figure 1, continued**

**
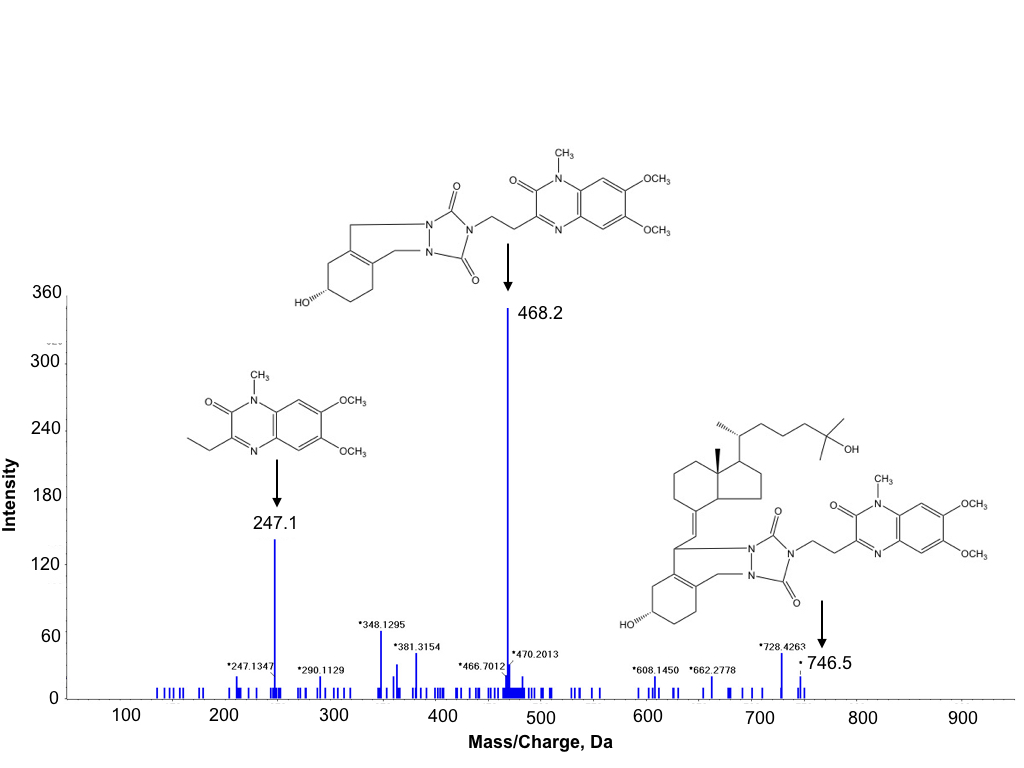
**

**B**

**
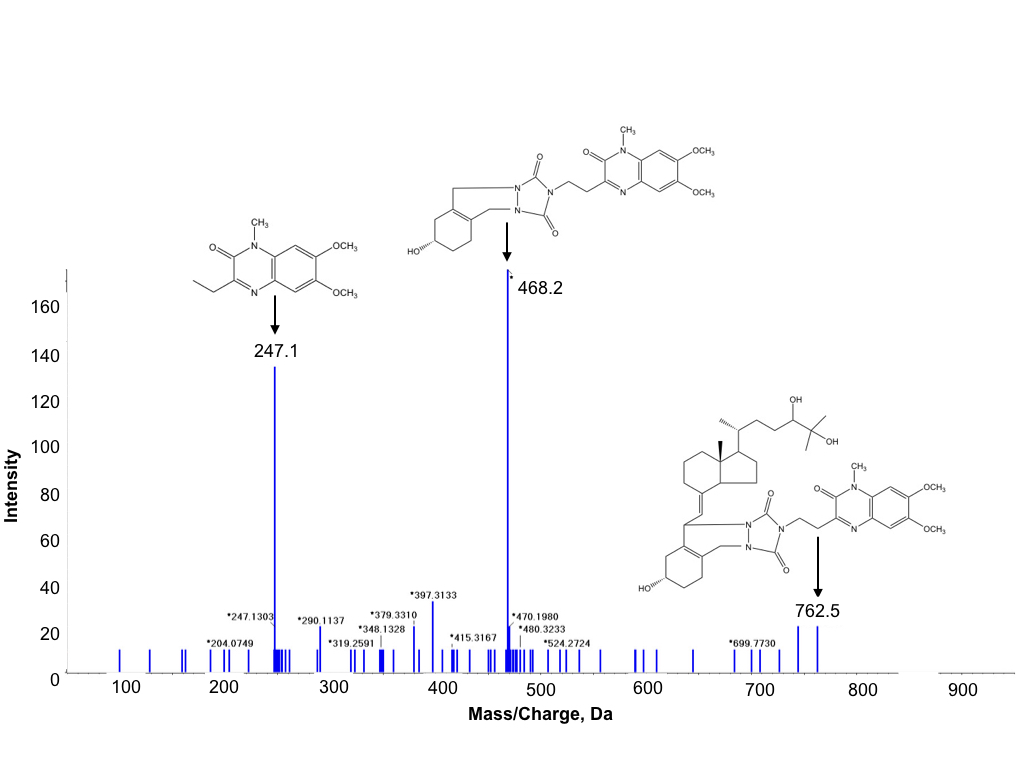
**

**Supplementary Figure 1, continued**

**
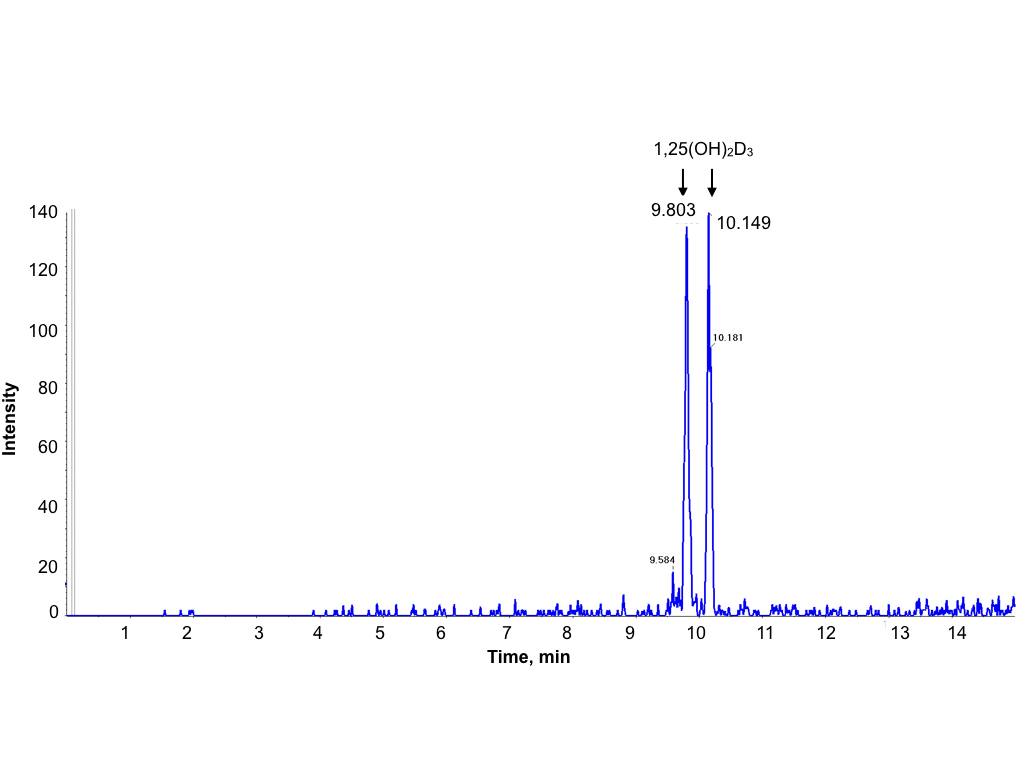
**

**C**

**
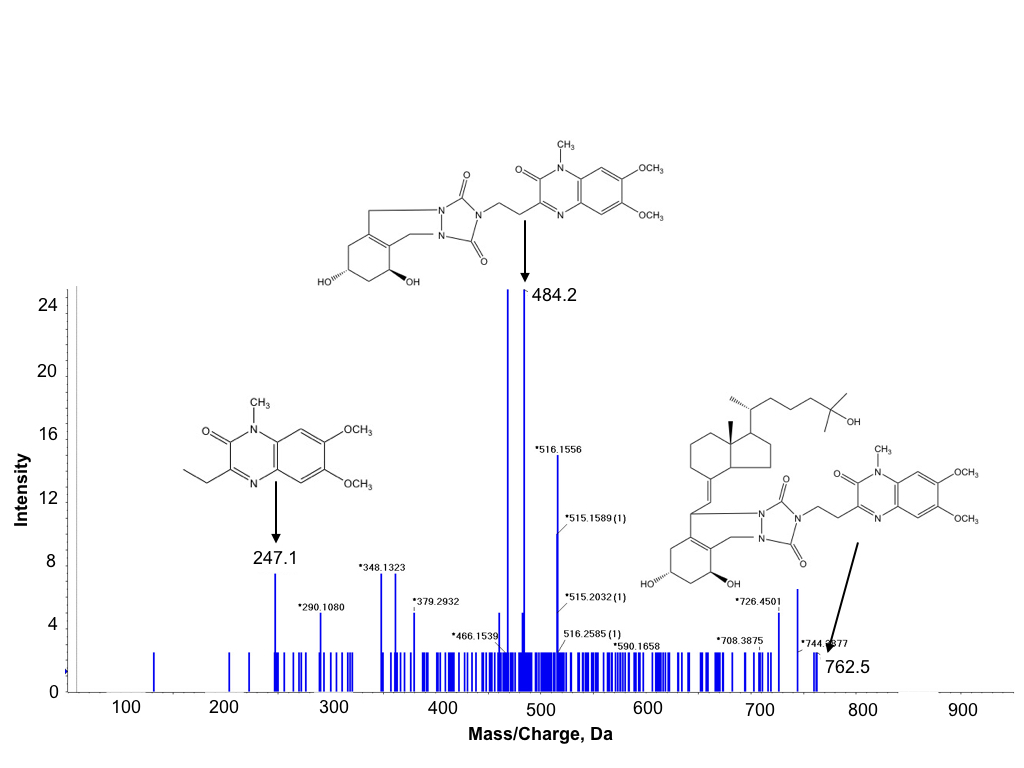
**

**Supplementary Figure 1. Chromatogram and fragmentation of DMEQ-TAD adducts of vitamin D, 25D, 24,25D, and 1,25D.** A) Composite chromatogram of multiple reaction monitoring for vitamin D, 25D, and 24,25D. B) Mass spectra for DMEQ-TAD adducts of vitamin D (730.5->468.2), 245D (746.5->468.2), and 24,25D (762.5->468.2), indicating that m/z=468.2 is the major fragment of vitamin D, 25D, and 24,25D ions. C) Chromatogram of multiple reaction monitoring for 1,25D, and mass spectra for DMEQ-TAD adduct of 1,25D (762.5->484.2), indicating m/z=484.2 is the major fragment of 1,25D ion. m/z=247.1 is the fragment of DMEQ-TAD.

**Supplementary Figure 2**


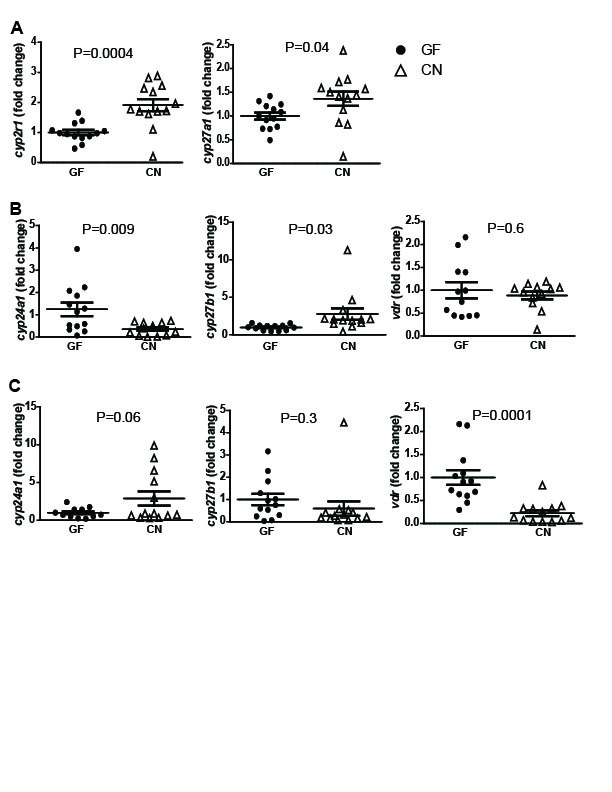


**Supplementary Figure 2. CN alters expression of mRNA for Cyp metabolizing enzymes** A) Kidney and B) colon expression of *cyp24a1*, *cyp27b1*, and *vdr*. C) Liver expression of *cyp24a1, cyp2r1* and *cyp27a1*. Data is expressed as fold change over GF expression levels. Values are the mean ± SEM of n=13 mice per group and combined data from 3 independent experiments. Student’s t-test was used to test significance.

**Supplementary Figure 3**

**
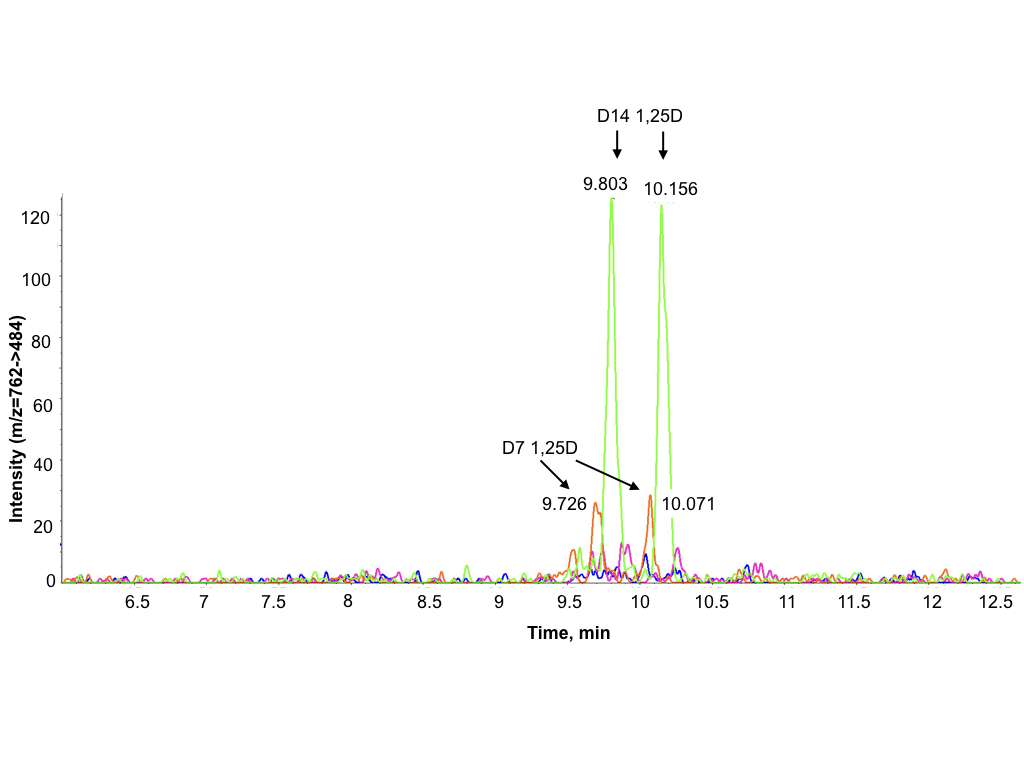
**

**Supplementary Figure 3. Chromatogram of multiple reaction monitoring 1,25D after CN**. Relative intensity of 1,25D from GF 0, 3, 7, and 14d after CN. D0 (blue) and 3 (pink) were not clearly distinguishable from background noise. Relative 1,25D was measured by LCMS, after using a 1,25D concentrating kit and the instructions provided (Immundiagnostick) as described.
